# Optimization of the Automated Synthesis of [^11^C]*m*HED—Administered and Apparent Molar Activities

**DOI:** 10.3390/ph12010012

**Published:** 2019-01-14

**Authors:** Chrysoula Vraka, Verena Pichler, Neydher Berroterán-Infante, Tim Wollenweber, Anna Pillinger, Maximilian Hohensinner, Lukas Fetty, Dietrich Beitzke, Xiang Li, Cecile Philippe, Katharina Pallitsch, Markus Mitterhauser, Marcus Hacker, Wolfgang Wadsak

**Affiliations:** 1 Division of Nuclear Medicine, Department of Biomedical Imaging and Image-guided Therapy, Medical University of Vienna, 1090 Vienna, Austria; chrysoula.vraka@meduniwien.ac.at (C.V.); neydher.berroteraninfante@meduniwien.ac.at (N.B.-I.); tim.wollenweber@meduniwien.ac.at (T.W.); anna.pillinger@meduniwien.ac.at (A.P.); maximilian.hohensinner@meduniwien.ac.at (M.H.); lukas.fetty@meduniwien.ac.at (L.F.); xiang.li@meduniwien.ac.at (X.L.); cecile.philippe@meduniwien.ac.at (C.P.); markus.mitterhauser@meduniwien.ac.at (M.M.); marcus.hacker@meduniwien.ac.at (M.H.); wolfgang.wadsak@meduniwien.ac.at (W.W.); 2 Department of Biomedical Imaging and Image-guided Therapy, Division of Cardiovascular and Interventional Radiology, Medical University of Vienna, 1090 Vienna, Austria; dietrich.beitzke@meduniwien.ac.at; 3 Institute of Organic Chemistry, University of Vienna, 1090 Vienna, Austria; katharina.pallitsch@univie.ac.at; 4 Ludwig-Boltzmann-Institute Applied Diagnostics, 1090 Vienna, Austria; 5 Center for Biomarker Research in Medicine, CBmed GmbH, 8010 Graz, Austria

**Keywords:** [^11^C]*meta*-hydroxyephedrine, radiosynthesis, separation, apparent molar activity

## Abstract

The tracer [^11^C]*meta*-Hydroxyephedrine ([^11^C]*m*HED) is one of the most applied PET tracers for cardiac imaging, whose radiosynthesis was already reported in 1990. While not stated in the literature, separation difficulties and an adequate formulation of the product are well known challenges in its production. Furthermore, the precursor (metaraminol) is also a substrate for the norepinephrine transporter, and can therefore affect the image quality. This study aims at optimizing the synthetic process of [^11^C]*m*HED and investigating the effect of the apparent molar activity (sum of *m*HED and metaraminol) in patients and animals. The main optimization was the improved separation through reverse phase-HPLC by a step gradient and subsequent retention of the product on a weakly-cationic ion exchange cartridge. The µPET/µCT was conducted in ten rats (ischemic model) and the apparent molar activity was correlated to the VOI- and SUV-ratio of the myocardium/intra-ventricular blood pool. Moreover, nine long-term heart transplanted and five Morbus Fabry patients underwent PET and MRI imaging for detection of changes in the sympathetic innervation. In summary, the fully-automated synthesis and optimized purification method of [^11^C]*m*HED is easily applicable and reproducible. Moreover, it was shown that the administered apparent molar activities had a negligible effect on the imaging quality.

## 1. Introduction

The world health organization (WHO) estimates that almost 18 million people die of cardiovascular diseases (CVD) each year. Moreover, from the year 2000 onwards, it has been reported that CVDs have become the number one cause of death worldwide [1,2]. Therefore, there is a high demand for a reliable imaging technique enabling the investigation of CVDs. One of the most frequently used positron emission tomography (PET) tracers in this regard is [^11^C]*meta*-hydroxyephedrine ([^11^C]*m*HED), also known as 3-[(1*R*,2*S*)-1-hydroxy-2-([^11^C]methylamino)propyl]phenol, a false transmitter agent and a norepinephrine transporter (NET) substrate [3,4]. It has been described that [^11^C]*m*HED underlies the uptake-1 pathway of NET from the synaptic cleft to the cytoplasm of the sympathetic nerve terminal. Advantages of [^11^C]*m*HED are the metabolic resistance against the enzymes MAO (monoamine oxidase) and COMT (catechol-O-methyltransferase), and in contrast to endogenous ligands, the high selectivity towards uptake-1, the low non-specific binding to the myocardium, the fast pharmacokinetics, as well as a continuous release and re-uptake by sympathetic nerves [5,6]. Thus, the combination of these biochemical properties, together with a fast and reliable automated radiosynthesis, allows PET acquisition duration of at least one hour, despite the short half-life of carbon-11 (20 min). Additionally, flow dependent effects can be examined when combined with a [^13^N]NH_3_ scan or duplex sonography [7]. In more recent studies, [^11^C]*m*HED has also been used to image the white-to-brown fat conversion, and it was shown that cold-induced BAT thermogenesis is controlled by the sympathetic nervous system [8,9]. Besides, [^11^C]*m*HED might be utilized in oncology for the diagnosis of adrenal tumors and metastasis [10,11].

The first [^11^C]*m*HED radiosynthesis was described in 1990 by Rosenspire et al. [4], which is still the most cited concerning radiosynthesis. Since then, only a few publications have dealt with the optimization of the radiosynthetic procedure. These studies focused mainly on automation and optimization, in terms of time efficiency and radiochemical yields. Indeed, producers of [^11^C]*m*HED know about the difficulties of HPLC separation and purification via solid-phase extraction, caused by the strong pH sensitivity of *m*HED. Nevertheless, this phenomenon has not been published. Furthermore, none of those investigations focused on the improvement of the molar activity or the reduction of the residual precursor concentration [3,5,12,13]. In fact, a blocking study demonstrated that the [^11^C]*m*HED precursor, metaraminol (*meta*-hydroxynorephedrine), can affect the image quality through displacement of the tracer itself. Consequently, the amount of residual metaraminol plays a major role in this matter. Besides the probability of triggering a pharmacological effect at high doses, both metaraminol and *m*HED are substrates for NET, and can therefore cause dose-dependent loss of image quality [14]. Thus, there is a necessity to include the sum of precursor and cold product concentrations (apparent molar activity) in the quality control criteria.

## 2. Results

### 2.1. Radiosynthesis of [^11^C]mHED

The radiosynthesis of [^11^C]*m*HED was set up on a GE Tracerlab FX C Pro and was performed fully automated. The methylation of metaraminol was highly reproducible in a mixture of DMF:DMSO as solvent, leading to a stable radiochemical yield of around 2–3 GBq. This yield was sufficient for 2–3 animals or at least 1 patient dose per production. The most common reason for a failed synthesis was the irregular reversed phase-high performance liquid chromatography (RP-HPLC) separation of metaraminol and [^11^C]*m*HED before radiosynthetic optimization. Changing the RP-HPLC purification to the buffer free step gradient led to a significantly more stable separation and improved reproducibility. Overall (see Table 1), 51 syntheses were performed with the reliable RP-HPLC set-up; out of these 51 syntheses 26 were used for pre-clinical and clinical studies, ten were incomplete production files or failed synthesis and were subsequently excluded. Fifteen radiosyntheses were solely performed for the purpose of experimentation with different conditions or testing the system. None of the failed syntheses was caused by separation problems.

For calculation of the molar activity, a calibration curve in the range of 0.1*–*5 µg/mL was prepared and the UV-Vis-signal was measured at 275 and 220 nm, respectively. For both wavelengths R^2^ was >0.99, whereas the slope was steeper at 220 nm, indicating a better sensitivity.

### 2.2. µPET/µCT Imaging

**Figure 1:**
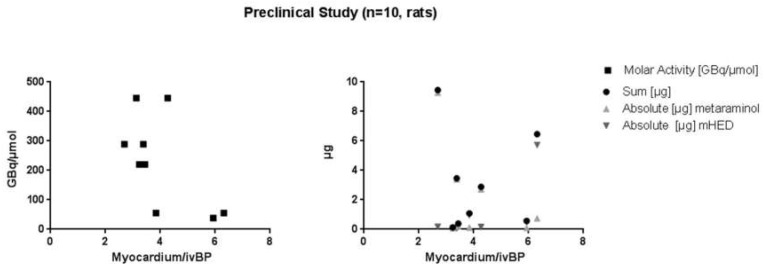
Correlation of the myocardium/intra-ventricular blood pool ratio and the molar activity, the mass of residual precursor and non-labelled *m*HED, as well as the sum of both.

The administered dose was 5 ± 8 µg/kg bodyweight for metaraminol and 2 ± 4 µg/kg for *m*HED, with an absolute mass dose of 2.3 ± 3.1 µg (sum of precursor and product), in a range of 0.1 to 9.5 µg (Table 2). No dose effects were visible in rats (Figure 1). Neither the correlation between the myocardium/intra-ventricular blood pool ratio and the molar activity, nor with the apparent molar activity or the sum of residual precursor and formed *m*HED showed any significance (*p*-value in all cases > 0.05).

**Figure 2:**
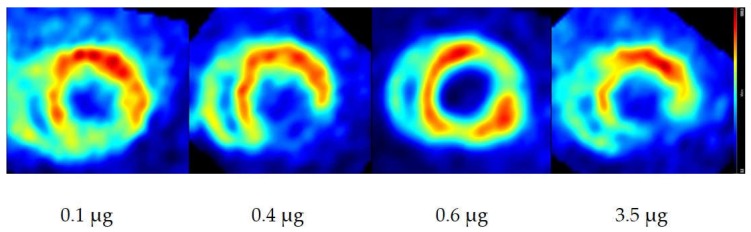
Representative cardiac images in short axis orientation of rat hearts (infarct model) with different applied mass doses of metaraminol and *m*HED. The sum of metaraminol and *m*HED is shown beneath the respective image.

For representative PET images see Figure 2 below. The ratios of myocardium/intra ventricular blood pool (Myo/ivBP) ranged between 3.1 and 6.3 for all animals. Infarcted areas are clearly definable within all applied masses.

### 2.3. PET/MRI Imaging

**Figure 3:**
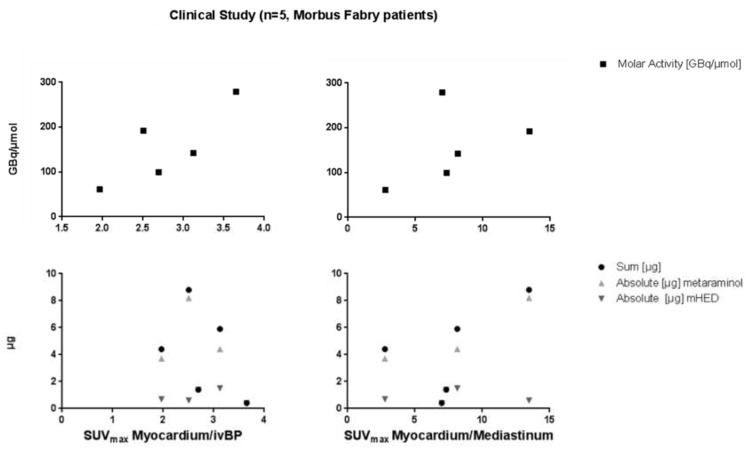
Correlation of the myocardium/intra-ventricular blood pool ratio (**left**) and correlation between the myocardium/mediastinum ratio (**right**) and the molar activity, the mass of residual precursor and non-labelled *m*HED, as well as the sum of both (Morbus Fabry patients).

No correlation was found between the myocardium/intra-ventricular blood pool ratio and the molar activity, or the overall mass of injected metaraminol and *m*HED (*p*-values ranged from 0.1 to 1) in Morbus Fabry patients (Figure 3). The same was true for the relation between the myocardium/mediastinum ratio and the mass or molar activity of the respective compound. Even expanding the analysis to ratios based on the SUV_mean_ did not lead to any correlation (data not illustrated). Only one of the five imaged Morbus Fabry patients showed a pathological finding. Excluding this patient from the correlation causes no changes in significance.

**Figure 4:**
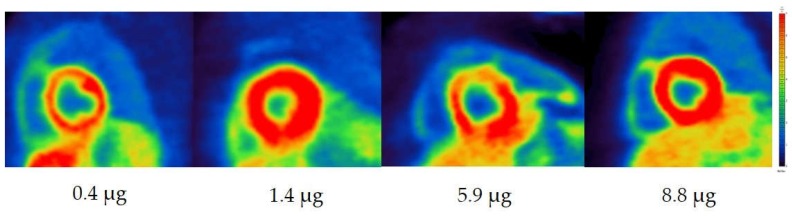
Cardiac images (short axis orientation) of Morbus Fabry patients with different applied mass doses of metaraminol and *m*HED. The sum of metaraminol and *m*HED is shown beneath the respective image. The patient with abnormality of sympathetic innervation was excluded in the figure, showing a Myo/Med ratio of <4 with an applied mass dose of 4.5 µg.

The ratio of myocardium/intra ventricular blood pool (Myo/ivBP), calculated from SUV_mean_ values, were in all cases 4 (ratio range for SUV_max_: 3 to 4). The myocardium/mediastinum ratio (Myo/Med) varies between 7 and 13 for the calculation, with SUV_max_ and SUV_mean_ (Figure 4).

**Figure 5:**
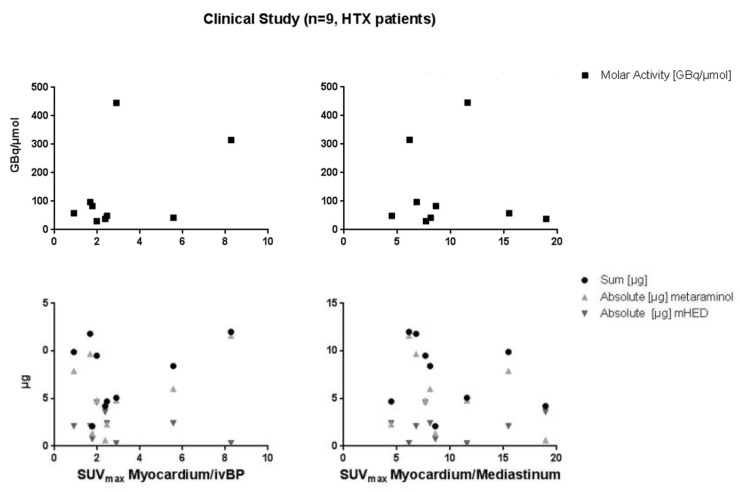
Correlation of the myocardium/intra-ventricular blood pool ratio (**left**) and correlation between the myocardium/mediastinum ratio (**right**) and the molar activity, the mass of residual precursor and non-labelled *m*HED, as well as the sum of both (heart transplantation (HTX) patients).

The correlations using the SUV_mean_ for the heart transplantation patients (HTX) showed again no significance for the Myo/ivBP (*p*-values between 0.3 and 0.6), as well as for the Myo/Med (*p*-values range from 0.4 to 0.6) ratios (data not illustrated). Moreover, no relationship was found using the ratios calculated with SUV_max_ values for the Myo/ivBP and Myo/Med versus the molar activity or the respective injected mass, or the sum of both compounds (*p*-value > 0.2, Figure 5).

## 3. Discussion

**Figure 6:**
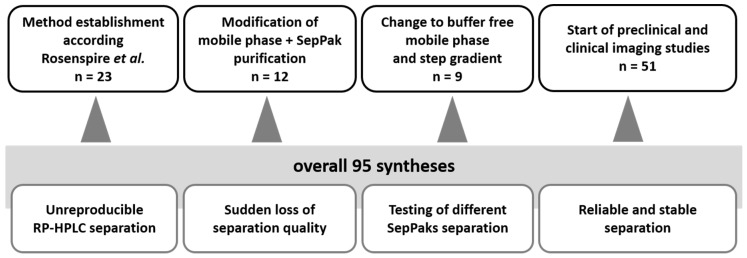
Overview on the radiosynthesis optimization, including the challenges and establishments during the first 44 syntheses and the stable separation process of the last 51 syntheses.

In general, [^11^C]*m*HED is a cardiac PET tracer with excellent pharmacokinetic properties, and can be used for diverse clinical issues like imaging of cardiac diseases, white-to-brown fat conversion, and oncology. The radiosynthesis is highly robust, as already reported in literature. Since the retention of *m*HED is strongly affected by minor variations in pH or salt concentration, a major challenge of the [^11^C]*m*HED radiosynthesis is the separation and purification from the precursor, side products, and the used methylation agent ([^11^C]CH_3_I or [^11^C]MeOTf). None of the published separation methods using semi-preparative RP-HPLC were reproducible or (long-term) repeatable in our laboratories (see Figure 6).

Changes in the separation efficiency occur even by necessary maintenance changes, such as renewing the column of a similar type. Almost 40 syntheses had to be performed to establish long-term stable conditions, and twelve out of these 40 syntheses were used to determine the purification potency via solid phase extraction (SPE). Here, different SPE cartridges were tested (XAD-4, C-18 or SCX), which showed either none or very strong retention of [^11^C]*m*HED. The product purification was achieved by removal of the mobile phase via a weakly-cationic ion exchange cartridge, and it was formulated in 3 mL of physiological saline.

The described synthesis procedure is now consistent and has been reproducible for over 50 preparations. None of the fail syntheses was due to poor separation or purification problems. The major achievement of this optimized procedure is the buffer free mobile phase and the utilization of the pH sensitivity of [^11^C]*m*HED to elute quickly from the RP-18 column caused by protonation.

Compared to previously published [^11^C]*m*HED productions, our method led to a reduced synthesis time, higher molar activities, and the apparent molar activity was discussed for the very first time (see Table 3). However, further comparison of the radiochemical yields or starting activities were not possible, due to insufficient information within the previously published radiosyntheses [5,12,15]. The overall administered doses for patients and animals was calculated in µg/kg bodyweight for metaraminol and *m*HED and correlated to the myocardium/intraventricular blood pool. To investigate the perfusion effect, a [^13^N]NH_3_ scan was performed upfront to the [^11^C]*m*HED imaging. However, neither patients nor animals suffered from pharmacological effects, nor any loss in imaging quality could be confirmed. The intra-individual variations and the progress of denervation had a higher impact on [^11^C]*m*HED accumulation than the apparent and molar activity. A limit of 50 nmol/kg (8.4 µg/kg) for metaraminol was stated to avoid effect on the imaging quality based solely on blocking studies, and the overall amount of metaraminol and *m*HED was not taken into account [14]. On the other hand, different doses of *m*HED were applied with constant concentrations of metaraminol in a recently published study. A washout or decreased time activity curve was observed from an applied concentration of 10 µg/kg bodyweight *m*HED [16]. The IC_50_ value of *m*HED towards NET drug inhibition was reported as 0.42 µM (competitive inhibition) by Foley at al. [17]. However, in PET studies, target binding affinities are required in the low nanomolar range to avoid competition to endogenous ligands. Therefore, the imaging mechanism of [^11^C]*m*HED is subject to long tissue life caused by several reuptake cycles, metabolic resistance, and rapid pharmacokinetics (Myo/ivBP ratio up to 5 after 10 min) [6,17]. With our new synthesis method, this limiting concentration was never reached, and by the correlation we could prove that higher doses of metaraminol, *m*HED, or the sum of both are necessary for observing impact on the image quality. Therefore, we could confirm that our administered amounts are too low for any effect on image quality or to trigger pharmacological effects.

## 4. Material and Methods

### 4.1. Radiosynthesis

**Figure 7:**
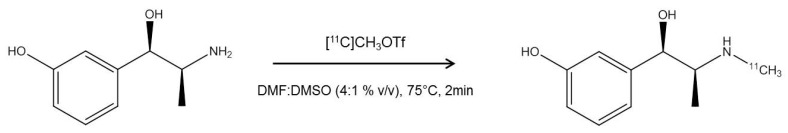
Reaction scheme of the radiosynthesis of [^11^C]*m*HED.

The [^11^C]CO_2_ was produced within a GE PETtrace cyclotron (General Electric Medical System, Uppsala, Sweden) by a ^14^N(p,α)^11^C nuclear reaction under irradiation of a gas target filled with N_2_ (+1% O_2_) (Air Liquide Austria, GmbH, Schwechat, Austria). The conversion of [^11^C]CO_2_ to [^11^C]CH_3_I was performed in the GE Tracerlab FX C Pro synthesizer. Therefore, the cyclotron production of [^11^C]CO_2_ was terminated at desired target activities between 67*–*127 GBq using a current of 45 µA for ~30 min, and the activity was trapped upon delivery on a molecular sieve (4 Å) within the synthesizer. Subsequently, [^11^C]CO_2_ was converted into [^11^C]CH_4_ by a Ni-catalyzed reduction with H_2_ at 400 °C. The [^11^C]CH_3_I was produced using gas phase conversion, as described in a previous study [18]. In short, the resulting [^11^C]CH_4_ was reacted with sublimated iodine at 738 °C in a recirculating process for 5 min to give [^11^C]CH_3_I. The produced [^11^C]CH_3_I was trapped on-line on a Porapak*^®^* N column and finally released by heating the trap to 190 °C. The [^11^C]CH_3_OTf was prepared on-line at the passage of [^11^C]CH_3_I through a silver triflate column (containing 300 mg impregnated graphitized carbon), pre-heated to 200 °C, at a flow rate of 40 mL/min. The [^11^C]CH_3_OTf was trapped in a glass reactor containing 1 mg metaraminol in 500 µL of a DMF:DMSO mixture (4/1 %*v/v*) and heated to 75 °C for 2 min (Figure 7). After the reaction time, the reactor was cooled to 35 °C and the reaction was quenched by adding 1 mL of RP-HPLC solvent (mobile phase A). The entire volume of the crude mixture was automatically transferred to a semi-preparative RP-HPLC column (see section semi-preparative scale purification). The [^11^C]*m*HED peak was collected in a round-bottom flask containing 10 mL of distilled water to increase the volume for subsequent solid-phase extraction, which was performed using a weakly-cationic ion exchange cartridge (Sep-Pak Accell Plus Short Cartridge, Waters [WAT020550]). After rinsing the cartridge with 10 mL water for complete removal of residual acetonitrile from the HPLC solvent, the pure product was eluted with 3 mL of 0.9% saline and filter sterilized (0.22 µm) under aseptic conditions (laminar air flow hot cell, class A).

### 4.2. Semi-Preparative Purification

**Figure 8:**
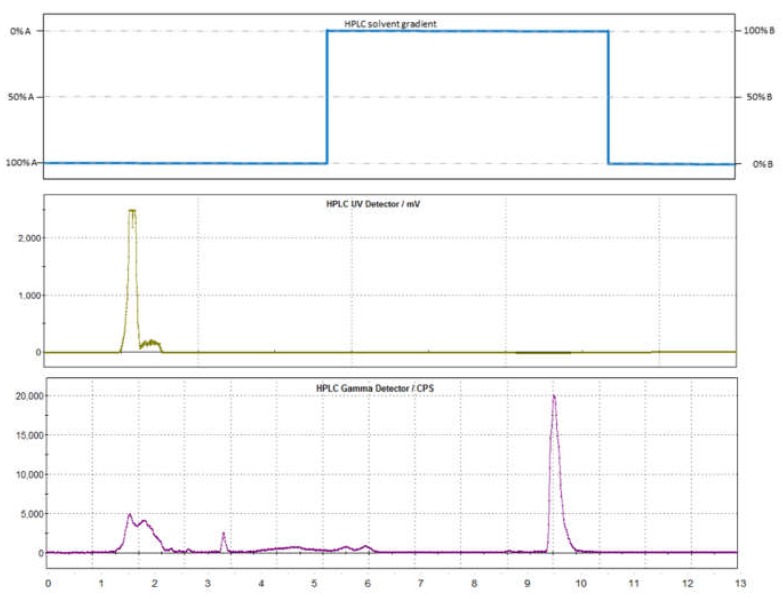
Typical chromatogram of the semi-preparative RP-HPLC purification of the crude mixture: The [^11^C]*m*HED elutes after 9.5 min from the C-18 RP column after using a step gradient, as illustrated at the top of the graphic. Solvent A consists of an acetonitrile:water mixture (58:42) and solvent B is a mixture of acetonitrile and acidified water (50:50; 0.004% H_3_PO_4_, conc).

Purification of the crude mixture was performed using a semi-preparative RP-HPLC (Supelcosil TMLC-ABZ+Plus, 5 μm, 250 × 10 mm (Supelco^®^, Bellefonte, PA, USA)) with a step gradient method. For the step gradient, two solvents were used: solvent A consisted of acetonitrile and water (58/42), and solvent B of a mixture of acetonitrile/water (50/50) mixture, which has been acidified (~pH 3, 0.1% H_3_PO_4_). The column was conditioned with solvent A, at a flow rate of 6 mL/min for injection, and then the flow was raised immediately to 8 mL/min. After the elution of [^11^C]CH_3_OTf, non-converted [^11^C]CH_3_I, precursor, as well as the DMF/DMSO mixture (approx. 6 min.), the mobile phase was switched to solvent mixture B. Subsequently, the [^11^C]*m*HED could be eluted with a retention time of 8–10 min (see Figure 8). After the completion of the RP-HPLC separation (after around 12 min), the mobile phase was set back to 100% solvent A to elongate column separation stability and lifespan, as long-time storage under acidic conditions should be avoided.

### 4.3. Quality Control

**Figure 9:**
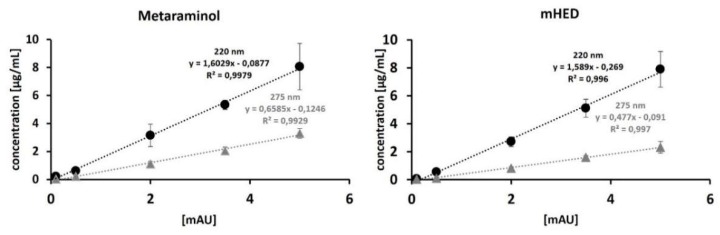
Calibration curve for metaraminol and *m*HED. Concentrations were chosen in the range of 0.1–5 µg/mL.

Radiochemical and chemical purities were assessed using analytical radio- and UV/Vis-RP-HPLC. Identity of [^11^C]*m*HED was confirmed by co-injection with the respective reference standard. In detail, an Agilent 1260 system (Agilent Technologies GmbH; Santa Clara, CA, USA) equipped with a quaternary pump (G1311B), a multi wavelength UV-detector (G1365D) set to 220 and 275 nm (reference wavelength 450 nm), a NaI (Tl) detector from Berthold Technologies (Bad Wildbad, Germany), and GINA Star controlling software (Elysia-Raytest; Straubenhardt, Germany) were used. As the stationary phase, an analytical RP-HPLC column, X-Bridge BEH Shield RP-18, 4.6 × 50 mm, 2.5 μm, 130 Å (Waters GmbH) was used [19]. Precursor and *m*HED concentrations were determined, and subsequently, the molar activity was calculated. The concentrations of the calibration curve were in the range of 0.1–5.0 µg/mL with R^2^ = 0.996 ± 0.002 (see Figure 9). The limit of detection for *m*HED was 0.22 µg/mL (220 nm) and 0.27 µg/mL (275 nm), and 0.23 µg/mL (220 nm) and 0.32 µg/mL (275 nm) for metaraminol. Sterility, absence of endotoxins, pH, osmolality, and residual solvents were determined by standard procedures routinely performed at the PET Centre of the Vienna General Hospital/Medical University of Vienna, and following similar monographs to the European Pharmacopoeia.

### 4.4. Animal Preparation

All procedures and protocols involving animals were conducted in compliance with and approval by the Institutional Animal Care and Use Committee of the Medical University of Vienna, Austria, as well as by the Austrian Ministry of Science, Research, and Economy (ZI. 1413/115-97/98 2014/2015). The manuscript adheres to the Directive European law (2010/63/EU) and to the ARRIVE guidelines for reporting animal experiments.

### 4.5. Imaging of µPET/µCT 

In vivo imaging experiments were conducted with a small animal-cone beam-computed tomography (µCT) and positron emission tomography (µPET) scanner (Siemens Inveon Multimodal μSPECT/µCT, dedicated μPET; Siemens Medical Solutions, Knoxville, TN, USA). Twelve to fourteen weeks old male Sprague Dawley rats (HIM:OFA, Himberg, Austria) underwent the scans 14–15 days after chirurgical intervention (myocardial infarction model), weighing 373 ± 45 g (n = 11), and were kept under controlled laboratory conditions (22 ± 1 °C; 12 h light/dark cycle), with food and water access ad libitum. Rats were anesthetized (1.5–2.0% isoflurane vaporized in oxygen 1.0–1.5 L/min) and positioned in the center of the field of view (FOV). Physiological parameters and the depth of anesthesia were constantly monitored and adapted throughout the experiment. All animals received [^11^C]*m*HED in 100% physiological saline (78 ± 12 MBq, molar activity: 211 ± 152 GBq/μmol (range: 38–446 GBq/μmol)). Once the radiotracer was administered through the lateral tail vein, µPET data acquisition took 30 min to allow tracer kinetics to attain full equilibrium. Three-dimensional static sinograms were generated from the list files, which were further reconstructed by an OSEM-3D/MAP algorithm (4 OSEM and 18 MAP iterations). All µPET data were corrected for attenuation, scatter, and decay. The image matrix was 256 × 256 pixels in the transversal plane. The µCT data were acquired within a full rotation and 360 projections with a FOV of 100 × 100 mm^2^. Furthermore, 80 kV, 500 µA, and 200 ms exposure time were used for the protocol settings. CT raw data was reconstructed with a Feldkamp algorithm, including ramp filtration and beam-hardening correction, resulting in a final image matrix of 1024 × 1024 pixels in the transversal plane. Data pre- and post-processing was performed by means of PMOD 3.8 (PMOD Technologies Ltd., Zurich, Switzerland). A 3D Gaussian filter with kernel size 1 mm × 1 mm × 1 mm was applied to the µPET data and a 2 × 2 × 2 reduction to the matrix was performed for the µCT data, in order to prevent excessive computing time. Rigid multimodal registration was performed to align both image datasets. A delineation of the volumes of interest (VOI), namely myocardium and intra-ventricular blood pool, was performed. We defined a good image quality, by the differentiability of myocardium and blood, respectively. Therefore, the ratio between myocardium/intra-ventricular blood pool was calculated. All animal data were excluded from the correlation when there was an incomplete data set available, due to technical problems or vitality of the animals.

### 4.6. PET/MRI Imaging

Institutional review board approval was gained for this prospective pilot study (IRB No.: 2301/2016 and 1562/2018). All patients gave written an informed consent prior to study inclusion. Patients were allowed to eat until 4 hours prior to the examination, but were asked to abstain from caffeine intake for 36 hours prior to PET/MRI imaging. None of the patients received medication that interferes with the presynaptic sympathetic nervous system (e.g., antidepressants, clonidine, etc.), beta-, or alpha blockade. All PET/MRI examinations (frames: 6 × 30 s, 2 × 60, 2 × 150, 2 × 300, 2 × 600) were conducted on a simultaneous PET/MR system (Biograph mMR; Siemens, Erlangen, Germany) according to a study published in 2013 [20]. Patients were positioned head first and supine. An ECG device was used for cardiac triggering. The [^11^C]*m*HED was injected through a venous cannula. A 40 min dynamic PET acquisition was performed for all patients.

All Morbus Fabry patients (n = 5) received [^11^C]*m*HED as well as [^13^N]NH_3_ for perfusion monitoring. Applied [^11^C]*m*HED activities were 320 ± 47 MBq, with molar activities of 155 ± 85 GBq/µmol (range: 61–279) formulated in 0.9% saline solution, and further diluted with saline to a standard volume of 17 mL. For the heart transplanted patients (HTX), the tracer formulation and scan procedure were the same, with applied activities of 470 ± 102 MBq (128 ± 148 GBq/µmol). From ten overall HTX patients, one had to be excluded due to a shorter scanning time. The myocardium/intra-ventricular blood pool ratio was calculated according to Bonfiglioli et al. [5].

### 4.7. Statistical Analysis

All data is shown as mean ± standard deviation, if not stated otherwise. The limit of detection was calculated according to ICH guidelines, and was defined as LOD = (3 × σ)/slope.

All ratios were correlated with the absolute applied concentration of *m*HED and metaraminol, the apparent and molar activity (pearson correlation, two-tailed *p*-value, and a confidence interval of 95%), and the *p*-values were determined using GraphPadPrism 6.01 (GraphPad Software 7825 Fay Avenue, Suite 230 La Jolla, CA 92037, USA).

## 5. Conclusions

Due to the optimized synthesis procedure of [^11^C]*m*HED, the overall synthesis time was reduced as well as the amount of the precursor metaraminol and *m*HED, and consequently a higher molar activity. Indeed, the beneficial feature is the buffer free RP-HPLC assay and the formulation through a weakly-cationic ion exchange cartridge. Therefore, a highly reproducible and repeatable, fully-automated radiosynthetic procedure could be developed. Furthermore, it was shown that the administrated molar and apparent activities are distinctly lower, and therefore no effect on the imaging quality was observed using this separation and purification method. Therefore, this robust production procedure is appropriate for preclinical as well as clinical studies without showing blocking or pharmacological effects in patients or animals.

## Figures and Tables

**Table 1:** Overview of overall synthesis data (excluding synthesis for the purpose of condition testing and fail synthesis), as well as synthesis data for the respective pre-clinical and clinical study.

[^11^C]*m*HED	Starting Activity [GBq]	Yield [GBq]	Yield [%EOB]	Molar Activity [GBq/µmol *m*HED]	Precursor Concentration [µg/mL]	*m*HED Concentration [µg/mL]
All synthesis (n = 32)	114 ± 15	3 ± 2	2.5 ± 1.5	126 ± 97	8 ± 7	3 ± 8
Patients:						
Morbus Fabry (n = 5)	124 ± 2	3.5 ± 0.4	2.9 ± 0.3	155 ± 85	6 ± 4	2 ± 1
HTX (n = 9)	120 ± 2	2.3 ± 1.3	2 ± 1	129 ± 148	5.5 ± 3.8	1.7 ± 2.8
Animals (n = 11)	122 ± 3	3 ± 2	2.4 ± 1.9	211 ± 152	4.6 ± 4.7	9 ± 19

**Table 2:** Overview on the applied mass concentrations of the precursor metaraminol and *m*HED as well as applied mass per bodyweight (BW). None of the patients received more than 0.1 µg/kg bodyweight of metaraminol and *m*HED, with a maximal administrated mass dose of 12 µg metaraminol and 4.6 µg *m*HED, respectively.

[^11^C]*m*HED	Metaraminol		*m*HED	
Patients	[µg/applied volume]	[µg/kg BW]	[µg/applied volume]	[µg/kg BW]
Morbus Fabry (n = 5)	5.2 ± 3.3	0.06 ± 0.05	1.6 ± 1.3	0.02 ± 0.02
HTX (n = 9)	5.5 ± 3.8	0.1 ± 0.1	2.1 ± 1.5	0.02 ± 0.02
Animals (n = 11)	1.8 ± 2.9	4.9 ± 8.4	0.8 ± 1.8	1.8 ± 3.8

**Table 3:** Comparison of radiosynthetic parameters of previously published and our optimized [^11^C]*m*HED method.

	Rosenspire et al. 1990	Nagren et al. 1995	Van Dort et al. 2000	Law et al. 2010	This work
Methylation agent	[^11^C]CH_3_I	[^11^C]CH_3_OTf	[^11^C]CH_3_I	According to Rosenspire et al.	[^11^C]CH_3_OTf
	250 µL DMF:DMSO (3:1)	100 µL ACN	anhydrous DMF 150 µL	250 µL DMF:DMSO (3:1)	DMF:DMSO (4:1, *v/v*%)
[c] Precursor	1 mg metaraminol free base, freshly prepared from metaraminol bitartrate	1 mg free base	0.6 mg free base	1 mg free base	1 mg free base
Formulation	10% EtOH + 0.24 M NaH_2_PO_4_, ~7 mL	physiological phosphate buffer 8 mL	0.04 M NaH_2_PO_4_ 10 mL	According to Rosenspire et al.	0.9% saline 3 mL
[c] Metaraminol	21 µg mean	Not reported	Not reported	2 µM	8 ± 7 µg/mL
[c] *m*HED		Not reported	Not reported	9 µM	3 ± 8 µg/mL
Radiochemical purity%	98 2% unidentified RI	Not reported	>98	95 ± 5	98 ± 2
Time of synthesis (EOB)	45 min without evaporation	Approx. 41 min	40 min	According to Rosenspire et al.	30–35 min
Molar activity (EOS) [GBq/µmol]	33 ± 18 (n = 19)	Not reported	47–60 (mean = 51) (n = 8)	10–30	126 ± 97 (n = 32)
